# Multi-organ Abnormalities and mTORC1 Activation in Zebrafish Model of Multiple Acyl-CoA Dehydrogenase Deficiency

**DOI:** 10.1371/journal.pgen.1003563

**Published:** 2013-06-13

**Authors:** Seok-Hyung Kim, Sarah A. Scott, Michael J. Bennett, Robert P. Carson, Joshua Fessel, H. Alex Brown, Kevin C. Ess

**Affiliations:** 1 Department of Neurology, Vanderbilt University School of Medicine, Nashville, Tennessee, United States of America; 2 Department of Pharmacology, The Vanderbilt Institute of Chemical Biology, Vanderbilt University School of Medicine, Nashville, Tennessee, United States of America; 3 Department of Pathology and Laboratory Medicine, University of Pennsylvania Perelman School of Medicine and Children's Hospital of Philadelphia, Pennsylvania, United States of America; 4 Department of Medicine, Vanderbilt University School of Medicine, Nashville, Tennessee, United States of America; Stanford University School of Medicine, United States of America

## Abstract

Multiple Acyl-CoA Dehydrogenase Deficiency (MADD) is a severe mitochondrial disorder featuring multi-organ dysfunction. Mutations in either the *ETFA*, *ETFB*, and *ETFDH* genes can cause MADD but very little is known about disease specific mechanisms due to a paucity of animal models. We report a novel zebrafish mutant *dark xavier* (*dxa^vu463^*) that has an inactivating mutation in the *etfa* gene. *dxa^vu463^* recapitulates numerous pathological and biochemical features seen in patients with MADD including brain, liver, and kidney disease. Similar to children with MADD, homozygote mutant *dxa^vu463^* zebrafish have a spectrum of phenotypes ranging from moderate to severe. Interestingly, excessive maternal feeding significantly exacerbated the phenotype. Homozygous mutant *dxa^vu463^* zebrafish have swollen and hyperplastic neural progenitor cells, hepatocytes and kidney tubule cells as well as elevations in triacylglycerol, cerebroside sulfate and cholesterol levels. Their mitochondria were also greatly enlarged, lacked normal cristae, and were dysfunctional. We also found increased signaling of the mechanistic target of rapamycin complex 1 (mTORC1) with enlarged cell size and proliferation. Treatment with rapamycin partially reversed these abnormalities. Our results indicate that *etfa* gene function is remarkably conserved in zebrafish as compared to humans with highly similar pathological, biochemical abnormalities to those reported in children with MADD. Altered mTORC1 signaling and maternal nutritional status may play critical roles in MADD disease progression and suggest novel treatment approaches that may ameliorate disease severity.

## Author Summary

Mitochondrial disorders have multiple genetic causes and are usually associated with severe, multi-organ disease. We report a novel zebrafish model of mitochondrial disease by inactivating the *etfa* gene. Loss of this gene in humans causes multiple acyl-Co dehydrogenase deficiency (MADD) that manifests with brain, liver, heart, and kidney disease. While presentations are variable, many children with MADD have a severe form of the disease that rapidly leads to death. We report that *etfa* gene function is highly conserved in zebrafish as compared to humans. In addition we uncovered potential disease mechanisms that were previously unknown. These include the impact of maternal nutrition on disease severity in their offspring as well as the role mTOR kinase signaling. Inhibition of this kinase with the drug rapamycin partially reversed some of the symptoms suggesting this may be a new approach to treat mitochondrial disorders.

## Introduction

Multiple acyl-CoA dehydrogenase deficiency (MADD), also known as glutaric aciduria type II (GA-II, OMIM #231680), is a rare autosomal recessive inherited metabolic disorder first described in 1976 [1]. The precise incidence and prevalence are unknown but are likely underreported given the variability in clinical presentation. MADD is caused by mutations in electron transfer flavoprotein genes A (*ETFA*), B (*ETFB*) or the ETF dehydrogenase (*ETFDH*) [2]. The *ETFA* and *ETFB* gene products, ETFα and ETFβ respectively, form an ETF heterodimer located in the mitochondria matrix [3]. This complex receives electrons from at least nine distinct dehydrogenases that are involved in fatty acid β-oxidation, amino acid and choline metabolism [4], [5], [6], [7]. Patients with MADD are classified by disease severity with type 1 having severe neonatal-onset with congenital anomalies, rapid deterioration and death [8]. Type 2 patients with MADD do not have congenital anomalies but still have a severe course with death usually during the few years of life [9]. Finally, type 3 patients have later onset and an overall milder course. However they still have hypoglycemia, metabolic acidosis, cardiomyopathy, hepatomegaly, kidney defects and neurological manifestations such as encephalopathy and leukodystrophy [10], [11].

Current treatments are mainly aimed at relieving symptoms though anecdotal reports of improvement after administration of riboflavin or Coenzyme Q have been reported [11]. While all types of MADD can be caused by *ETFA*, *ETFB* or *ETFDH* mutations, it is not understood why there is such variability in disease severity. Several reports indicate a marked buildup of fatty acids, amino acid or toxic compounds in multiple organs in patients with MADD. However, comprehensive cellular and molecular analyses have not been possible as there are no animal models available that recapitulates the spectrum of abnormalities seen in patients with MADD. The first animal model of MADD was created by inactivating the zebrafish *etfdh* gene [12]. This mutant zebrafish was named *xavier* (*xav*) with conserved metabolic abnormalities also observed in MADD patients including increased levels of acylcarnitines and glutaric acid. However *xav* mutant zebrafish did not recapitulate morphological defects observed in MADD patients. This may be due to early lethality seen in this model prior to later stages of organogenesis.

Using forward genetic screening for mutants with abnormal livers, we identified a mutant zebrafish called *dark xavier* (*dxa^vu463^*, termed hereafter as *dxa*) due to its phenotype of a dark fatty liver and hepatomegaly. *Dxa* mutant zebrafish have a nonsense mutation in the *etfa* gene resulting in widespread abnormalities broadly similar to those observed in MADD patients. We found large increases of acylcarnitines and glutaric acid in *dxa* mutants associated with multiple abnormalities of various organs including brain, liver, kidneys and heart. Marked accumulation of neutral lipid drops including cerebroside sulfate and free cholesterol in multiple organs was also observed. Analyses by mass spectrometry [13] found a large increase in triacylglycerides in *dxa* mutants but also a significant decrease of phosphatidylserine species which was also observed in human tissue derived from a patient with MADD [14]. The multiple defects seen in *dxa* mutant zebrafish closely recapitulate many core abnormalities observed in human patients with MADD. Interestingly, *dxa^vu463^* mutant developed hyperplasia with increased cell size in multiple organs including brain, liver and kidney suggesting activation of mTORC1 signaling. Excessive maternal feeding also exacerbated the phenotype in *dxa* mutants. We confirmed that mTORC1 signaling is highly elevated in *dxa* with increased phosphorylation of S6 and 4E-BP1. Treatment of *dxa* zebrafish with rapamycin alleviated a subset of signaling and cellular proliferation abnormalities suggesting that targeting mTORC1 signaling could be a rational therapeutic approach for patients with MADD.

## Results

### Identification and Classification of *dxa^vu463^* Mutant Zebrafish and Worsened Phenotype after Maternal Overfeeding

**Figure 1:**
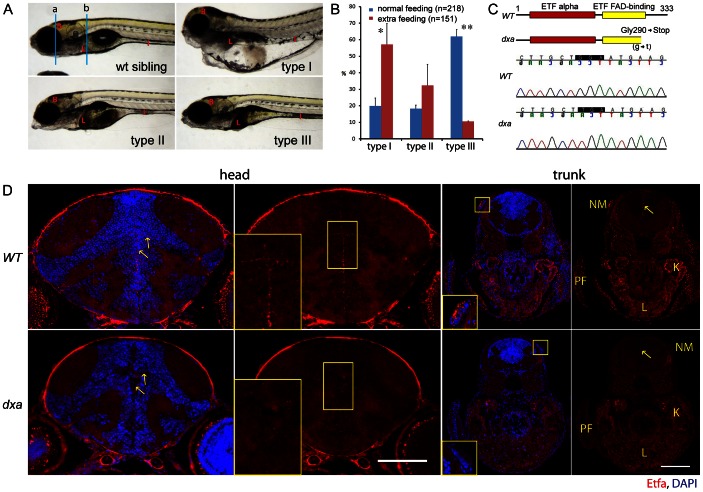
Classification of *dxa^vu463^* homozygous mutants, positional cloning of *dxa^vu463^* and Etfa protein expression. (A) Representative phenotypes of most severe (type I), moderate (type II) and mild (type III) *dxa* homozygous mutants at 7 dpf. Blue lines (a and b) indicate region of transverse sections in D. (B) Spectrum changes of type I, II and III mutants under different feeding conditions. Blue bars indicate the proportion of mutants under regular feeding (n = 218, 5 clutches), red bars for the proportion of mutants under extra feeding condition (n = 151, 3 clutches), p* = 0.03, p** = 0.00015. (C) Primary predicted structure of Etfa protein in wild-type and *dxa* zebrafish. Shaded codon indicates the null mutation of *etfa* in *dxa* zebrafish (GGA (Glycine) to TGA (stop)). (D) Anti-Etfa immunostaining (red) in wild-type control (upper panel, n = 9/9) and homozygous mutant (lower panel, n = 9/9) at 9 dpf. DAPI (blue) was used for nucleus staining. Arrows indicate Etfa expression in the ventricular region of the brain. Magnified midline views of yellow rectangles are in the left corners. Magnified rectangles on the trunk sections indicate neuromast hair cells. NM, neuromast; PF, pectoral fin; K, kidney; L, liver. Scale bar = 100 µm.

We identified *dxa* mutants during a forward genetic screen using ENU mutagenized zebrafish. Homozygous *dxa* mutants had a large and dark colored liver at 7 days post fertilization (dpf) (Figure 1A). However, when more closely examined, *dxa* mutants had a broad spectrum of defects during development and post developmental stages (Figure 1A). About 20% of mutants had severe congenital defects (type I) that included a small head and cardiac edema, these larvae died by 5–6 dpf. Approximately 18% of mutants were type II with moderate defects including an abnormal head and dark liver, intestine and brain. These died by 7–8 dpf. The remainder of the mutants (approximately 62%) classified as type III had mild defects that were morphologically close to wild type zebrafish except for a darker appearing liver, intestine and brain (n = 218). Type III mutants lived for 10 dpf in the unfed state whereas control siblings live for 10–12 dpf. Overall, type I, II and III mutants accounted for approximately 25% of the total zebrafish in each cross suggesting the *dxa* phenotype was due to a defect in an autosomal recessive gene. This was later confirmed (see below) as a mutation in the *etfa* gene known to be involved in mitochondrial function. Given the potential for metabolic influences on mitochondrial disease, we studied whether maternal overfeeding prior to egg laying could influence the phenotype. One week of extra feeding caused a dramatic shift in severity with 57% of *dxa* mutant zebrafish now classified as type I, 32% type II and 11% type III (n = 151) (Figure 1B). This result suggests that the maternal nutritional state dramatically affects the severity of *dxa* zebrafish and may also explain similar phenotypic variability reported in patients with MADD.

### Positional Cloning of the *dxa^vu463^* Mutation and Expression Analysis of Zebrafish *etfa* Gene

To identify the mutant gene in *dxa* zebrafish, we performed conventional linkage mapping and were able to map the likely gene to approximately 0.18 cM from the *galk2* gene, located on zebrafish chromosome 25 (1/547 recombination, data not shown). Whole genome sequencing of *dxa^vu463^* and control zebrafish ultimately identified a mutation in the *etfa* gene approximately 360 kb from *galk2*. This G to T mutation introduces a premature stop codon (G290X) in *etfa* (Figure 1C). Zebrafish *etfa* has 80% homology to the human *ETFA* gene suggesting a highly conserved function (sequence alignment not shown). Whole mount *in situ* hybridization of e*tfa* mRNA shows maternal and ubiquitous expression during early development with subsequent high expression levels maintained in the midbrain and blood vessels at 30 hours post fertilization (hpf) as well as liver and pectoral fins at 2 dpf (Figure S1A). Immunofluorescent staining with an anti-Etfa antibody also revealed high expression of Etfa protein in neural progenitor cells located adjacent to the ventricles of the brain of wild type larvae (Figure 1D, head, inset). We also saw strong expression in neuromast hair cells as well as kidney, liver and skeletal muscle of the pectoral fin of wild type at 9 dpf (Figure 1D, trunk, inset, n = 9/9). However, negligible Etfa protein was detected in *dxa* zebrafish (Figure 1D, bottom panel, n = 9/9. There is some residual signal in the *dxa* zebrafish that we interpret as non-specific binding of the secondary antibody to the outer pial membranes of the brain and outer eye (Figure 1D). Immunoblot analyses also detected a very minimal amount of Etfa protein in the *dxa* mutant (Figure S1C). This also supports a loss of function mutation due to non-sense mediated decay of *etfa* mRNA given the location of the premature stop codon in exon 10 and the *in situ* expression data (Figure 1D lower panel, Figure S1B. 10/10). This expression pattern of *etfa* further supports an important role in high energy demanding cell types such as neural progenitors within the brain, hepatocytes and kidney tubule cells.

### Increased Acylcarnitine and Glutaric Acid Profiles in the *dxa^vu463^* Mutant Zebrafish Recapitulates That Seen in Patients with MADD

While genetic testing of affected patients is ideal, MADD can be strongly suspected in symptomatic children who exhibit increased serum acylcarnitines and glutaric aciduria [7]. Using tandem mass spectroscopy to determine acylcarnitine levels, we found significantly higher level of multiple long-, medium- and short-chain acyl-CoA species and isovalerylcarnitine in the *dxa* mutant larvae compared to control siblings (Figure S2A). This suggests that dysregulation of mitochondrial β-oxidation is highly similar in *dxa* to that observed in patients with MADD. Further analysis of organic acids using gas chromatography-mass spectrometry found approximately 6.5 µg of glutaric acid per *dxa* larvae (Figure S2C), but no detectable amount seen in control siblings (Figure S2B). This pattern is highly reminiscent of that seen in patients with MADD, also known as glutaric aciduria Type II (GA-II).

### *etfa* Mutation Causes a Severe Mitochondrial Defect with Progressive Accumulation of Triacylglycerol and Free Cholesterol in the Liver

**Figure 2:**
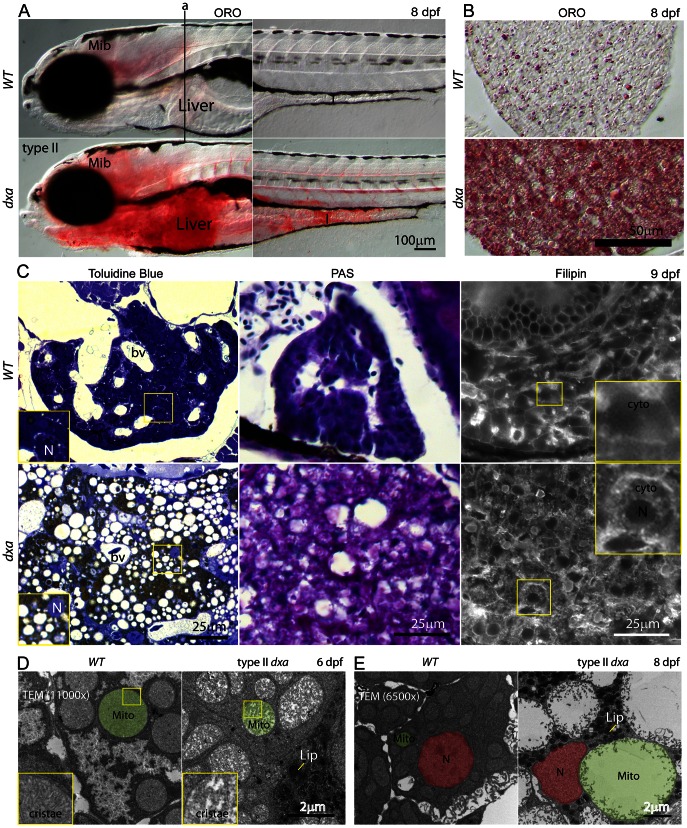
Lipids, cerebroside sulfate and free cholesterol accumulations in the cytosol of type II *dxa^vu463^* hepatocyte. (A) Whole mount Oil Red-O (ORO) staining of wild type and type II *dxa* at 8 dpf. Vertical line (a) indicates location of transverse section in B. (B) ORO staining in the liver sections at 8 dpf. (C) Toluidine blue, PAS and Filipin staining at 9 dpf. Wild type control livers are on the top row and *dxa* are on the bottom row. Magnified views of rectangles showing toluidine blue are in the lower left corners. The brown colored drops in Toluidine blue staining suggests cerebroside sulfate accumulation. Magnified views of rectangles showing Filipin (free cholesterol) staining are in the upper right corners. Filipin appears to accumulate in the cytosol of mutant hepatocytes. (D) TEM image at 6 dpf. Green shadows mark single representative mitochondria. Magnified views of rectangles showing cristae are on the left lower corners. (E) TEM image at 8 dpf. Nuclei are colored red and single representative mitochondria are again colored green. Dark granules in *dxa* mutants appear to represent lipid drops. Mib, midbrain; I, intestine; L, liver; bv, blood vessels; N, nucleus; cyto, cytosol; Mito, mitochondria; Lip, lipid drops. Scale bar = (A) 100 µm, (B) 50 µm, (C) 25 µm and (D, E) 2 µm.

Hepatic steatosis is a central sign in MADD, likely resulting from defective fatty acid β-oxidation that may be exacerbated during episodes of hypoglycemia. *Dxa* mutants exhibit progressive accumulation of dark colored granules in multiple organs including brain, liver and intestine after 6 dpf (see Figure 1A). Oil Red O (ORO) staining in whole mounts of type II *dxa* mutant larvae and coronal sections showed massive accumulations of neutral lipid in the brain, liver and intestine as well as blood vessels at 8 dpf (Figure 2A, B). Interestingly, toluidine blue staining of thick sections used for Transmission Electron Microscopy (TEM) revealed many heterogeneous sized vacuoles in the liver with brown colored drops in the cytosol of hepatocytes (Figure 2C). As glycosphingolipid can include glucose or galactose and sulfate groups (cerebroside sulfate) that can be stained with toluidine blue, the brown drops within hepatocytes may contain cerebroside sulfate. Intensive Periodic Acid Schiff (PAS) staining was also seen in *dxa* liver (Figure 2C, middle) suggesting that these lipid drops are comprised of cerebroside sulfate as liver glycogen is normally undetectable in unfed 8 dpf zebrafish. Additional support that the drops do not contain glycogen is supported by TEM analyses where we did not observe any glycogen containing granules at 6 or 8 dpf (data not shown). We also found high levels of free cholesterol in the cytosol of *dxa* hepatocytes using filipin staining (Figure 2C, right). However, we did not see lipid and free cholesterol accumulation in *dxa* liver at 6 dpf although the mutants already exhibit hepatomegaly and enlarged hepatocytes (Figure S3A–D)

Enlarged cell size was a consistent phenotype at later stages (Figure S3E, F). *Dxa* hepatocytes were approximately three times larger those seen in control siblings (Figure S3G). These results suggest that intrinsic abnormalities of hepatocytes led to both lipid and cholesterol accumulation in *dxa* mutant zebrafish. We then analyzed cellular ultrastructure using TEM to investigate possible organelle defects. The internal mitochondrial cristae density was markedly decreased in type II *dxa* hepatocytes at 6 dpf although total mitochondrial size was not changed (Figure 2D). Strikingly, we found extremely large mitochondria with minimal cristae in type II mutants just 2 days later at 8 dpf (Figure 2E). From these TEM results, we conclude that the “vacuoles” we saw in the mutant liver are actually grossly swollen mitochondria (Figure 2C, E). This suggests that Etfa is required for mitochondrial maintenance as well as energy metabolism. We assessed mitochondrial function in *dxa* mutants by measuring oxygen consumption over time. We found significantly decreased oxygen flux in *dxa* mutant zebrafish compared to sibling controls (Figure S4). This strongly supports an impairment of mitochondria function in *etfa* mutant cells.

### Mitochondrial Defects in *dxa^vu463^* Kidney Epithelium

**Figure 3:**
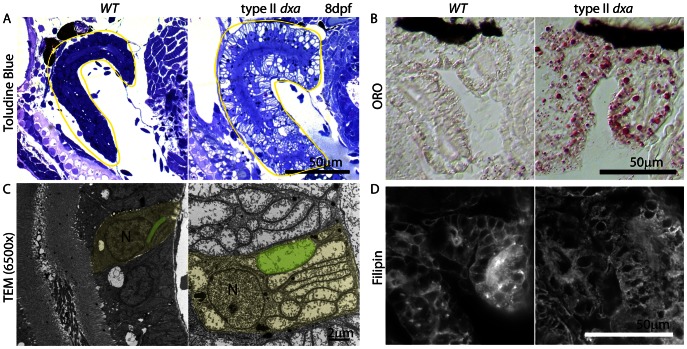
Kidney defects in 8 dpf *dxa^vu463^* mutant zebrafish. (A) Toluidine blue staining in pre-TEM sections at 8 dpf. Various sized vacuoles were observed in the *dxa* mutants. (B) ORO staining showing marked increase in lipids in *dxa* mutants. (C) TEM of kidney epithelium in wild type (left) and *dxa* mutants (right). Green colored regions indicate rod-shaped mitochondria in the kidney. (D) Filipin staining again shows free cholesterol accumulation in the cytosol of kidney cells. N, nuclei. Scale bar = 50 µm (A, B, D) and 2 µm (C).

It has been reported that many patients with severe neonatal onset MADD have polycystic kidney disease though Bohn et *al.* and Harkin et *al.* reported that these kidneys were pathologically distinct from typical polycystic kidney disease [15], [16]. We did note high Etfa expression within pronephric tubules of wild type kidneys (Figure 1D, trunk). Histological analysis of toluidine blue stained sections of *dxa* zebrafish kidney showed clear abnormalities possibly resulting from hypertrophy of the pronephric tubular epithelium. We also found a large number of prominent vacuoles in both type II and III *dxa* kidney epithelium compared to wild type (Figure 3A). TEM analysis of type II *dxa* mutants showed similar to hepatocytes, they are very large mitochondria with minimal cristae (Figure 3C, 2E). This finding implies that the “cystic kidney” pathology ascribed to patients with MADD may be due to massively swollen mitochondria in kidney tubule cells. We further found lipid and free cholesterol accumulation in the cytosol of *dxa* mutant kidney cells (Figure 3B, D). However, we did not see significant increases of lipid and free cholesterol in *dxa* mutants that have only mild defects at earlier stages, though hypertrophic kidney tubule cells with swollen mitochondria are already present.

### Increased Number of Neural Progenitor Cells and Accumulation of Neutral Lipid and Cerebroside Sulfate in the *dxa^vu463^* Brain

**Figure 4:**
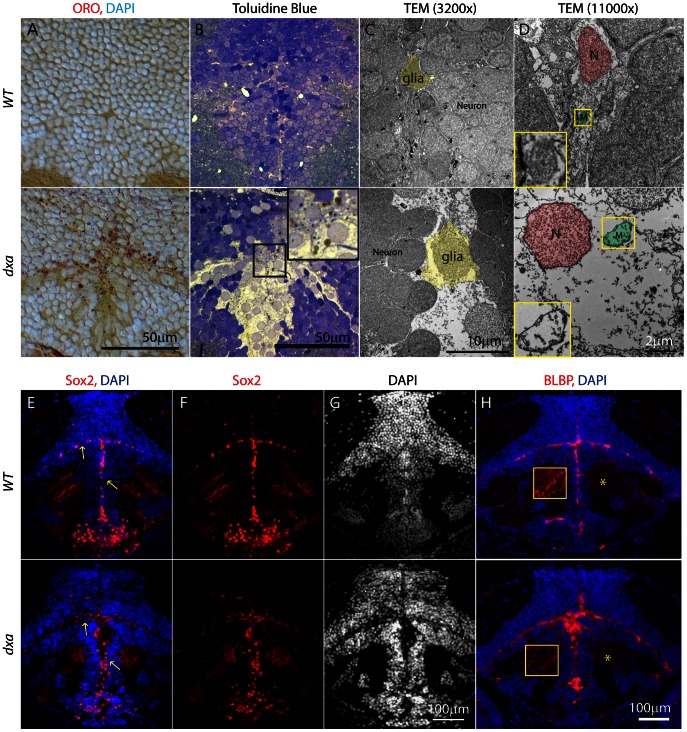
Lipid accumulation and necrotic features in the *dxa^vu463^* mutants with increased numbers of neural progenitor cells and dysmorphic brain. Top panels show control wild type zebrafish with bottom panels showing images from type II *dxa* mutant at 8 dpf. (A) ORO (red) showing increased lipids in the mutant brain, DAPI (bright blue) staining nuclei. (B) Toluidine blue staining in wild type and type II *dxa* with severe brain defects. Magnified view of midline region in the rectangle is shown on the upper right corner with greatly enlarged neural progenitor cells and neurons. Brown colored vesicles again suggest lipid drops containing cerebroside sulfate. (C) TEM image of VZ in *WT* (top) and type II *dxa* (bottom). Yellow pseudocolor indicates a single glia cell in *WT* and *dxa* mutants, marked increase in cell size is present. (D) Higher magnification image of neural progenitor cells. Green pseudocolor region indicates individual mitochondria. Pseudocolor with red indicates nuclei. Magnified views of normal and mutant mitochondria are shown on the left lower bottom. (E) Anti-Sox2 (red) and DAPI (blue) staining in control (top) and *dxa* zebrafish (bottom). Yellow arrows indicate Sox2 positive cells in the VZ. (F) Red channel image of (E). (G) DAPI channel of (E) showing disrupted gray matter of brain. (H) Anti-BLBP staining in wild type (top) and *dxa* (bottom). Asterisks indicate white matter region normally containing glia fibers. Region within the yellow box is further magnified to allow fine details of glial fibers to be seen. Contrast levels of control (top) and *dxa* zebrafish (bottom) were adjusted together to compare glial fibers. Scale bar = 50 µm (A, B), 10 µm (C), 2 µm (D) and 100 µm (E–H).

ORO staining also revealed extensive lipid drops in the brain (Figure 2A). We then analyzed brain sections to more precisely determine the location and cell types that contain lipid. We found large lipid accumulation in the ventricular zone (VZ) of the brain where the neural progenitor cells are found (Figure 4A). Within the same region of *dxa* mutant brain, we also found cerebroside sulfate containing lipid drops at 9 dpf (Figure 4B). Interestingly, type II mutants with more severe defects have VZ cells with very large nuclei compared to other neurons, but these large cells had pale intracellular staining (Figure 4B). TEM revealed that these cells do not have a discernible subcellular structure other than swollen nucleus and mitochondria (Figure 4C, D). These findings are suggestive of ongoing necrosis although we could not identify ruptured cell membranes. Of note we did not see enlarged nuclei at 6 dpf but swollen mitochondria were still observed in the type II mutant brain (data not shown). These results suggest progressive and rapid brain damage after 6 dpf in *dxa* zebrafish.

To analyze whether those abnormal cells are neural progenitors, we performed immunostaining for Sox2 in both type II and III *dxa* larvae at 8 dpf. We found increased numbers of Sox2 positive cells in both type II and III mutants (n = 9/9). Statistical analysis of type II mutants showed a 75% increase of Sox2 positive cells in the dorsal part of the VZ at 8 dpf (Figure S5). Finally, we found that brain lipid-binding protein (BLBP) positive neural progenitor cells were increased and distorted in their morphology though had decreased processes within the white matter of *dxa* mutants (Figure 4H, asterisk and yellow magnified inset, n = 6/6). Altogether, these results suggest that neural progenitor cells in the *dxa* mutant are hyperplastic and hyperproliferative. They may also be unable to properly generate neurons given the abnormal appearing grey matter observed in *dxa* mutant brain (Figure 4).

### Lipidomic Analysis of *dxa^vu463^* Shows Increase of TAG but a Decrease of Phosphatidylserine

Given the overt increases in lipids seen by Oil Red O staining, we performed lipid profiling with mass spectrophotometry (MS) to identify differences of lipid molecular species between control and *dxa* mutant larvae at 8 dpf. We found moderately decreased monoacylglycerol (MAG) and diacylglycerol (DAG) in the *dxa* mutant though only the MAG decrease was statistically significant. However a large increase of triacylglycerol (TAG) was observed in the *dxa^vu463^* mutants (Figure S6).

Using MS analyses of glycerophospholipids, we also found significant decreases in phosphatidylserine (PS) species (Figure S6). By contrast, the two most abundant phospholipid species phosphatidylcholine (PC) and phosphatidylethanolamine (PE) did not show any statistically significant differences between control and *dxa* mutant zebrafish. These results provide a rationale for future lipid modifying therapies in patients with MADD and will help focus future experiments on lipid abnormalities seen in *etfa* mutant zebrafish.

### Facial Axon, Mechanosensory Hair Cell, and Myelination Defects in *dxa^vu463^* Mutant

**Figure 5:**
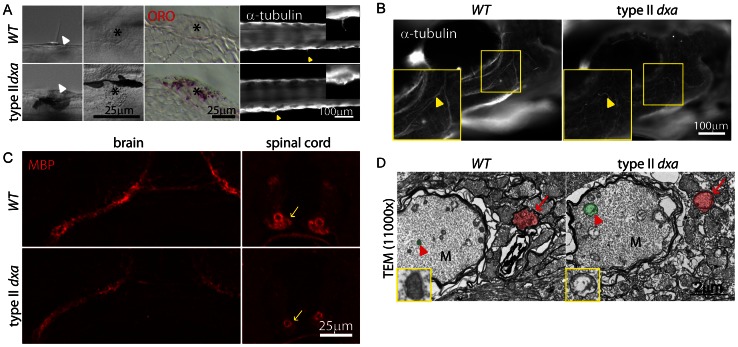
Facial axons, mechanosensory hair cell and myelination defects in type II *dxa^vu463^* mutant zebrafish. (A) DIC imaging of live cilia (first column) and neuromast cells (second column, asterisks), ORO stained lipids in neuromast cells (third column, asterisks). Acetylated-tubulin marks cilia (yellow arrowheads). Magnified views of cilia are shown on the upper right corner. (B) Whole mount immunofluorescence staining of acetylated-tubulin in WT (left) and *dxa* mutants (right) at 8 dpf. Yellow arrowheads indicate facial axons. Rectangle region is magnified in lower left corner. (C) anti-MBP staining in the brain (left) and spinal cord (right) in WT (top) and *dxa* mutant zebrafish (bottom) at 8 dpf. Arrows indicate myelinated axons in the spinal cord, all signal is reduced in *dxa* mutant zebrafish. (D) TEM (11,000×) image of spinal cord as indicted by arrows in C. Normal (WT) and swollen (*dxa* mutant) mitochondria are pseudocolored green, indicated by large red arrowhead). Red arrows indicate less condensed myelination layer in a *dxa* axon. Further magnified views of mitochondria are on the lower left corner. M, Mauthner axon track. Scale bars are as indicated in each panel.

High Etfa expression was found within neuromast cells that are zebrafish sensory organs (Figure 1D). Interestingly, type I and II *dxa* mutants had a decreased response to touch stimulation (data not shown) that was correlated with the severity of defects and increased age. Neuromast cells had short or absent kinocilia in type II *dxa* mutant and using ORO staining we found the dark granules seen in *dxa* neuromasts are comprised of lipids (Figure 5A, seen in 10/10 mutant larvae examined). Given these widespread lipid abnormalities in sensory structures, we also looked for alterations in sensory nerves tracts. Using acetylated tubulin, we noted decreased staining with a disorganized appearing, “kinked” axonal track in type II *dxa* mutants (Figure 5B, n = 8/9). We then examined expression of myelin basic protein (MBP), the most abundant myelin associated protein in the brain and spinal cord. We found decreased MBP staining in *dxa* type II mutants (Figure 5C, n = 6/6) but did not see significant changes in type III mutants (data not shown, n = 5/6). Using TEM to examine myelination in the spinal cord, we again found abnormally increased size and morphology of mitochondria in the Mauthner axon track (sensory pathway mediating escape responses) as well as decreased myelination (Figure 5D). These hypomyelination findings are reminiscent of the leukodystrophy reported in patients with MADD [17].

### Rapamycin Sensitive and Insensitive mTORC1 Signaling in *dxa^vu463^* Mutant Zebrafish

**Figure 6:**
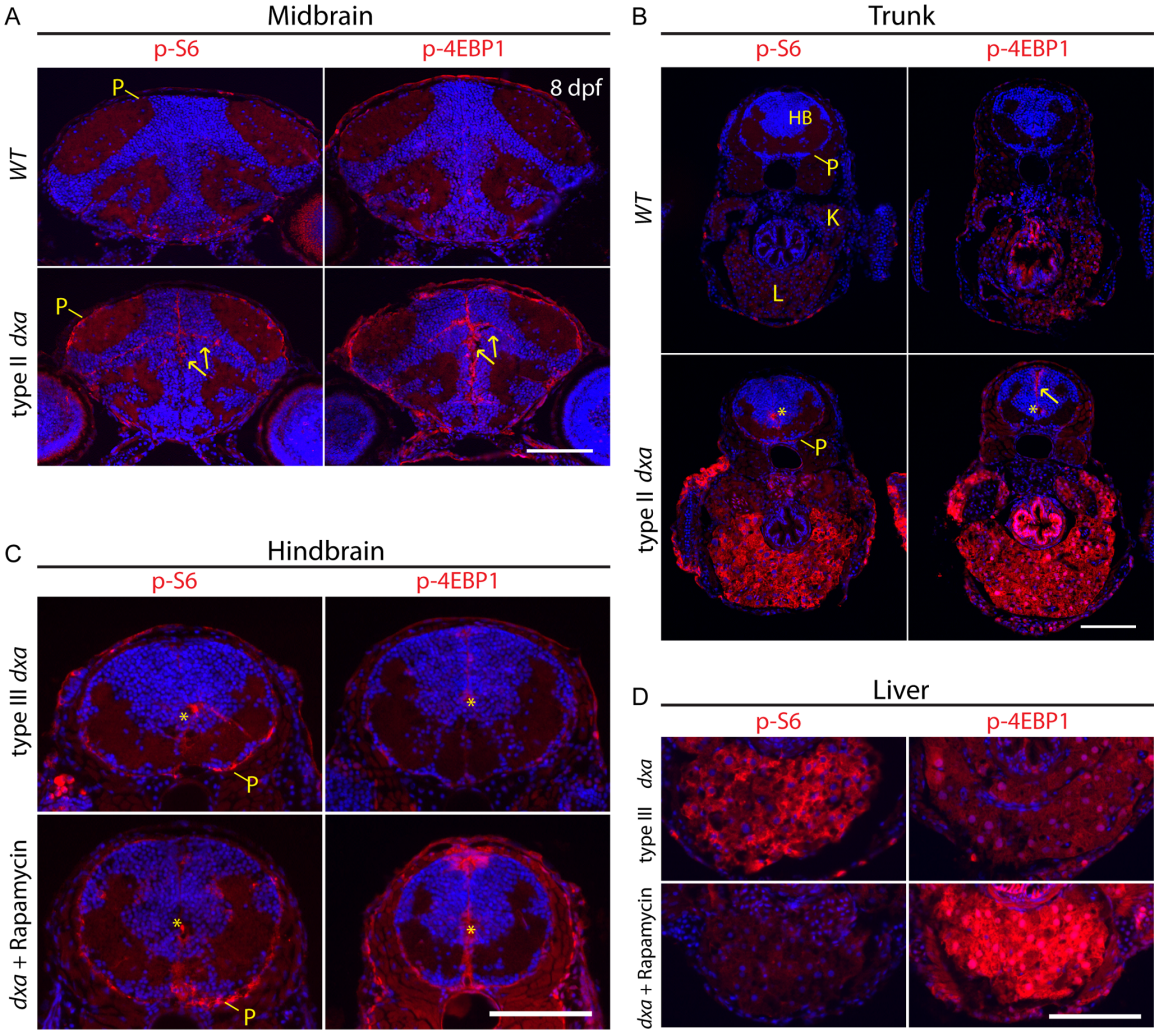
Tissue dependent regulation of mTORC1 activation in *dxa^vu463^* mutant zebrafish. Anti-phospho-S6 (left panels) and anti-phospho-4E-BP1 (right panels) antibodies were used to assess mTORC1 kinase activity. (A) WT brain (top) and *dxa* brain (bottom) at 8 dpf. Arrows indicate p-S6 and phospho-4E-BP1 positive cells in neural progenitors of the brain. P-S6 and p-4E-BP1 are also detected in the superficial pial cells of the mutant brain. (B) Sections of trunk regions in WT (top) and *dxa* (bottom) at 8 dpf. Phospho-S6 was detected in the central canal and phospho-4E-BP1 positive cells were found central canal as well as midline cells (yellow arrow) in *dxa* mutant zebrafish. Asterisks indicates central canal of hindbrain. 300 nM of rapamycin was used from 5 dpf to 8 dpf to treat *dxa* mutant zebrafish in C and D. (C) Hindbrain regions of type III mutant at 8 dpf. Phospho-S6 and phospho-4E-BP1 staining was again detected in central canal (*) and pial cell sheath (P) in both control and rapamycin treated *dxa* mutants. DAPI (blue) was used for nuclei staining. (D) Liver regions of same sections seen in (C) with marked suppression of phospho-S6 but a relative increase in phsohp-4E-BP1 levels. P, pial cell sheath; HB, hindbrain; K, kidney; L, liver. Scale bar = 100 µm.

The markedly enlarged cells in *dxa* mutant brain, liver and kidney suggests that mTORC1 could be involved as signaling through this kinase is a key controller of cell size [18]. We previously showed activation of this pathway in zebrafish causes increased cell size that can be reversed with rapamycin, a potent mTORC1 inhibitor [19]. We then evaluated the phosphorylation status of the mTORC1 downstream effectors S6 ribosomal protein and 4E-BP1 by immunofluorescence. We found markedly elevated phospho-S6 and phospho-4E-BP1 in *dxa* mutants especially in neural progenitor cells (n = 4/6 (phospho-S6), 6/6 (phospho-4E-BP1)) and pial cells (n = 6/6 (phospho-S6), 6/6 (phospho-4E-BP1)) on the midbrain and central canal of the hindbrain (Figure 6A, B). Increased level of phospho-4E-BP1 was seen more broadly than phospho-S6 in *dxa* mutants notably within midline cells and the central canal of the hindbrain as well as the intestine (Figure 6B). Immunoblots of *dxa* mutants also revealed increased mTORC1 signaling compared to control larvae at 6 dpf (Figure S7A–C). We also found increased phospho-S6 and phospho-4E-BP1 in the kidney and liver at 8 dpf (Figure 6B). Even at earlier time points before the pathology was overt, we still found increased mTORC1 signaling in the liver (Figure S7D). Given these findings we hypothesized that mTORC1 inhibition could potentially rescue *dxa* mutants. However, rapamycin treatment from 3–9 dpf was not able to suppress the *dxa* mutant phenotype (data not shown). To address whether activated mTORC1 observed in *dxa* mutants is rapamycin sensitive, we treated with rapamycin daily from 5 dpf to 8 dpf at a concentration of 300 nM. Rapamycin freely crosses the blood brain barrier in zebrafish and typically inhibits mTORC1 downstream completely [19]. In contrast to results we have obtained with other zebrafish models of human disease, we found that levels of phospho-S6 and phospho-4E-BP1 were not fully suppressed in the brains of treated larvae (Figure 6C). In the liver rapamycin did in fact suppress phospho-S6 levels but phospho-4E-BP1 actually appeared to be increased (Figure 6D) [20]. This intriguing finding suggests a novel regulation of mTORC1 downstream effectors in *dxa* mutant zebrafish.

**Figure 7:**
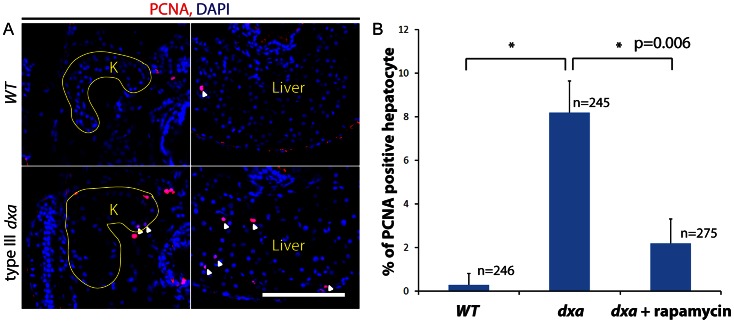
Increased proliferation in *dxa^vu463^* mutant kidney and liver. (A) PCNA staining in the kidney and liver of wild type (top) and type III *dxa* mutant (bottom). Yellow lines indicate kidneys. DAPI (blue) was used for nuclei staining. (B) PCNA positive cells were counted from three different *WT*, *dxa* mutant and rapamycin treated *dxa* mutant liver section. N = 246 (WT), 245 (*dxa*), 275 (*dxa* + rapamycin) cells. *p = 0.006 for both comparisons.

We previously found that *tsc2* mutant zebrafish with prominent mTORC1 activation had both increased cell size and increased proliferation [19]. To assess proliferation in *dxa* mutants, we analyzed the proportion of kidney and liver cells expressing proliferating cell nuclear antigen (PCNA) at 8 dpf. Very few PCNA positive cells were detected in control siblings but a highly increased proportion of cells express PCNA in type III *dxa* mutant at 8 dpf (Figure 7A). We quantified these differences in the liver and found 1/246 PCNA positive cells in control larvae versus 20/245 in type III *dxa* mutant zebrafish, this difference was statistically significant, p<0.006 (Figure 7B). We then treated with rapamycin from 5 to 8 dpf to verify if suppression of mTORC1 signaling could rescue aspects of the *dxa* phenotype. While mutants treated with rapamycin still developed a fatty liver, there was a clear decrease in cellular proliferation (Figure 7B). This result suggests that mTORC1 may be considered as a potential therapeutic target for some of the pathological features seen in patients with MADD.

## Discussion

MADD is a complex genetic disease with multi-organ involvement and widespread biochemical abnormalities. These features likely reflect the impairment of multiple acyl-CoA dehydrogenases with each enzyme normally handling different substrates. Additional MADD complexity may be due to distinct mutations in *ETFA*, *ETFB* or *ETFDH*. While patients with *ETFDH* mutations predominate in the literature, this is possibly due to a bias of genetic testing for relatively milder forms of MADD that are compatible with longer survival. Patients with *ETFA* mutations in contrast may have a more severe course and rapidly succumb to this disease prior to an accurate clinical, biochemical and genetic assessment. MADD is now screened in newborns in many countries and the true prevalence of all genotypes should eventually emerge from prospective analysis of confirmed positive cases.

Comprehensive analysis of MADD features and pathological mechanisms including genotype/phenotype relationships has been severely hampered by the lack of genetic animal models that recapitulates key features of MADD. In this study we analyzed a novel zebrafish model with a loss of function mutation of the *etfa* gene. Remarkably, *dxa* zebrafish recapitulates many key MADD features including biochemical abnormalities, a phenotypic spectrum from severe (type I and II) to moderate (type III) and multi-organ defects of the brain, liver and kidney. The shared phenotype of hepatic steatosis and dysmorphic kidneys seen in patients with MADD and *dxa* mutant zebrafish are likely due to defects of fatty acid β-oxidation as well as disruptions of amino acid and choline metabolism. C4 (butyryl) and C5 (isovaleryl) acylcarnitines and glutaric acid were highly elevated, this confirms the remarkable conservation of zebrafish and human mitochondrial function. However we did note species-specific differences. For example, patients with MADD have elevations of C14:1 but we also observed increases of fully saturated C16 and C18 in zebrafish. This suggests that the substrate availability for very long chain acyl-CoA dehydrogenase (VLCAD) in the zebrafish diet differs from the human fatty acid pool and that zebrafish primarily oxidize saturated fatty acids.

In contrast to MADD/GAII, GA Type I is caused by mutation in glutaryl-CoA dehydrogenase (GCDH), however this is one of dehydrogenases coupled to the ETF complex. *Gcdh* knockout mice did not have any obvious brain defects, but on a high lysine diet, these mice had neuronal loss, defective myelination and swollen mitochondria [21], [22]. Though abnormalities of neural progenitor cells were not reported, their results suggest that accumulation of glutaric acid may be sufficient to cause defective myelination and mitochondrial abnormalities although the clinical differences between GA-I and GA-II support distinct pathological mechanisms for each disease. Strikingly, we found the severity may be caused by the nutritional state of the parents as extra feedings prior to egg fertilization produced a much higher proportion of type I and II mutants in each cross. Ongoing studies in our laboratory will investigate whether this mechanism is due to additional metabolic “stress” or from alterations of key maternal proteins, lipids or mRNA in the yolk. However, our findings indicate that a better understanding of nutrition and overfeeding may positively impact fetuses with MADD and could reduce severe congenital anomalies. The low frequency of this disease and the lack of prenatal diagnosis in families without a previously diagnosed proband makes this scenario unlikely but given the trend towards precise genetic diagnoses for all aspects of medicine, maternal diet potentially exacerbating the MADD phenotype may be a crucial finding.

mTORC1 signaling is a key mediator of cell size control and differentiation. Using other zebrafish models of human disease, rapamycin treatment reversed abnormalities of cell size and mTORC1 signaling in the brain [19]. In marked contrast, brain abnormalities and other aspects of mTORC1 signaling in *dxa* zebrafish appeared to be rapamycin resistant. Phospho-S6 was entirely inhibited in the liver of *dxa* zebrafish but levels of phospho-4E-BP1 were actually elevated by rapamycin. It was previously shown that rapamycin inhibits phosphorylation of S6 and 4E-BP1 differentially [20]. This group reported that S6K and S6 phosphorylation were readily abolished throughout the duration of rapamycin treatment but phosphorylation of 4E-BP1 can recover despite initial inhibition and repeated application of rapamycin. We do not understand the mechanism leading to rapamycin resistant mTORC1 signaling in the brain and liver but speculate it may be related to increased amino acids in *dxa* zebrafish that could activate Rag proteins [23]. Increased leucine for example is sufficient to cause Rag GTPase dependent translocation of mTORC1 to lysosomes [24]. Isovaleric Co-A dehydrogenase requires the ETF complex and loss of function mutations in the *ISOVALERYL-CoA DEHYDROGENASE (IVD)* gene are known to cause accumulation of isovaleric acid, a metabolite of leucine [25], [26]. Leucine accumulation in *dxa* may then be activating mTORC1. We found markedly increased leucine levels in *dxa* larvae supporting this potential mechanism (Figure S8). We also found markedly increased p62/sequestosome 1 in *dxa* mutant zebrafish (data not shown) that was recently shown to be essential to activate mTORC1 [27]. These findings suggest that restricting intake of leucine and other branched amino acids may be important in MADD to suppress symptoms due to mTORC1 activation. However, increased aerobic glycolysis was observed in *etfdh* mutant zebrafish, this may compensate for a failure of mitochondrial beta oxidation [12]. Increased glycolysis may provide key intermediates for cell proliferation [28] and elevated mTORC1 signaling could further increase glycolysis by modulating transcription of genes required for this metabolic process [29]. This may represent a compensatory mechanisms and inhibition of mTORC1 with rapamycin could exacerbate the MADD phenotype or precipitate a metabolic crisis. We have seen no evidence for this in our animal model but caution against the use of mTORC1 inhibitors in patients with MADD outside of well regulated clinical trials.

The myelination defects in *etfa* mutant zebrafish are notable given the severe neurologic deficits including encephalopathy that is usually seen in patients with MADD. Lysosomal disorders such as metachromatic leukodystrophy (MLD) [30], Krabbe disease [31] and Gaucher disease [32] all have accumulation of cerebroside sulfate that appears to cause myelination defects in nerve system as well as hepatomegaly. We also see accumulation of cerebroside sulfate in radial glia and hepatocytes in *dxa* mutant larvae. It is possible that inhibition of autophagy by mTORC1 activation might contribute to symptoms in MADD. The markedly increased p62 levels in *dxa* mutants supports such a mechanism.

In conclusion, we report the first animal model of MADD due to mutations of the *etfa* gene. *Dxa* mutant zebrafish larvae have an array of biochemical and pathological features that strongly indicates this is a relevant model for MADD. *Dxa* zebrafish can be effectively employed to generate and test further hypotheses about disease specific mechanisms. In addition, *dxa* mutant zebrafish will be invaluable for future *in vivo* chemical screens to identify therapeutic compounds that may ameliorate disease aspects of MADD and potentially other mitochondrial disorders.

## Materials and Methods

### Fish Strains

Zebrafish strains used in this study included AB* and *dxa^vu463^*. Embryos were obtained from natural matings and raised at 28.5°C in egg water (0.3 g of sea salts/L). For overfeeding experiments, we gave an extra meal of TetraMin Tropical Flakes daily for one week prior to fertilization of eggs. The normal diet is twice a day meal of brine shrimp and Tropical Flakes Monday through Friday and once day on Saturday and Sunday of each week. Short term extra feeding does not cause any obvious phenotypes. 5 pairs of heterozygous siblings were used for this experiment. We fed normally one week and each pair of each was mated. Then we gave the same zebrafish extra food for the subsequent week and mated again. This cycle was repeated three times.

### Whole Mount *In Situ* Hybridization

Antisense digoxigenin-labeled RNA probe for etfa was produced using a DIG-RNA labeling kit (Ambion). Embryos were fixed in 4% paraformaldehyde overnight, and dehydrated in 100% methanol at −20°C. Whole mount hybridization was performed using standard protocols [33]. BCIP/NBT (Vector laboratories) mixture was used as a chromogenic substrate. *In situ* images were acquired using a Zeiss Axioscope and Nikon Coolpix 4500 digital camera.

### Immunofluorescence Staining

To avoid staining variation, 3 control and 3 *dxa* mutant larvae were processed together in the same slide glass. Slides were processed in a Sequenza Slide Rack. Embryos were fixed in 4% paraformaldehyde from overnight to two days at 4°C. Fixed embryos were embedded in 1.2% agarose/5% sucrose and saturated in 30% sucrose at 4°C for 1 to 2 days. Tissue blocks were frozen in 2-methyl butane. 10 µm sections were collected on microscope slides using a Leica cryostat. Sections were kept in −80°C before use. Sections were rehydrated in 1× PBS and blocked in 5% sheep serum in PBS for 2 hours, they were then incubated with primary antibodies to Etfa (Genetex, #GTX124324, dilution 1∶300), Sox2 (abcam, #97959, dilution 1∶500), PCNA (Sigma, #P8825, dilution 1∶3000), BLBP (abcam, #ab32423, dilution 1∶500), phospho-S6 ribosomal protein (Cell Signaling #2215 Ser235/236, dilution 1∶300), and phospho-4E-BP1 (Cell Signaling #2855 Thr37/46, dilution 1∶300) overnight at 4°C, washed 10 minutes×3 times with 1× PBS and then incubated for 2 hours with Alexa Fluor conjugated goat anti-rabbit secondary antibodies. Sections were then washed with 1× PBS for 30 minutes and mounted in Vectashield with DAPI (Vector laboratories). Antigen retrieval for PCNA staining was performed for 30 minutes of boiling in 10 mM sodium citrate before blocking. Images were acquired using Zeiss Axiovert 200M microscope with Zeiss AxioCam MRm and Hamamathu digital camera. Digital images were processed using Adobe Photoshop CS5 and Adobe illustrator CS5. All images received only minor modifications with control and mutant sections always processed in parallel.

### Whole Mount Immunofluorescence Staining

Fixed samples were rinsed with PBS-DT (1× PBS, 0.5% Triton X-100, 2% DMSO) and both control and mutant were incubated with blocking solution (PBS-DT, 5% goat serum) for 2 hours at room temperature in a single tube. The antibody against acetylated-tubulin (Sigma, #T7451, dilution 1∶500) was used overnight at 4°C. Larvae were rinsed with PBS-DT 3 times (10 minutes each). Secondary was a goat Cy3-anti-mouse for overnight at 4°C. Specimens were rinsed with 700 µL of PBS-Tween for 10 minutes and repeated 5 times. Zebrafish were fixed in 4% PFA and mounted in glycerol before being imaged.

### Oil Red O Staining

For whole mount staining at larvae stage, larvae were fixed in 4% PFA overnight. Control and *dxa* mutant larvae were rinsed three times (5 minutes each) with 1× PBS/0.5% Tween-20 (PBS-Tween). After removing PBS-Tween, larvae were stained with mixture of 300 µL of 0.5% ORO in 100% isopropyl alcohol and 200 µL of distilled water for 15 minutes. Larvae were then rinsed with 1× PBS-Tween for three times. Larvae were rinsed twice in 60% isopropyl alcohol for 5 minutes each. They were briefly rinsed in PBS-Tween and fixed in 4% PFA for 10 minutes. Larvae were mounted in glycerol prior to imaging. For high resolution ORO staining on transversely sectioned larvae, 10 µm sections were dried at room temperature for 5 minutes. 150 µL of working ORO solution was added to slides and stained for 30 seconds. They were then washed with tap water and mounted using Vectashield with DAPI.

### Filipin and Periodic Acid-Schiff (PAS) Staining

For free cholesterol staining on transversely sectioned larvae, slides were soaked with 1× PBS for 5 minutes, then Filipin complex diluted 1∶500 (Sigma, F-976) was added directly to slides and stained for 1 minute in the dark. Slides were washed with PBS and mounted with 75% glycerol. Images were taken using the DAPI channel of a fluorescent microscope.

Frozen sections were used for PAS staining. The PAS stain was conducted in the Translational Pathology Core laboratory at Vanderbilt University using a DAKO Artisan Link Staining System.

### Lipid Analysis

Glycerophospholipids from zebrafish larvae were extracted using a modified Bligh and Dyer procedure [34]. Forty of 8 dpf larvae of each genotype, either mutant or sibling control were homogenized in 800 µl of ice-cold 0.1 N HCl∶CH_3_OH(1∶1) using a tight-fit glass homogenizer (Kimble/Kontes Glass Co, Vineland, NJ) for about 1 minute on ice. The suspension was then transferred to cold 1.5 mL Eppendorf tubes and vortexed with 400 µl of cold CHCl_3_ for 1 min. Centrifugation (5 minutes at 4°C, 18,000× g) to separate the two phases. Lower organic layer was collected, an odd carbon internal standard was added and solvent evaporated. The resulting lipid film was dissolved in 100 µl of isopropanol∶hexane∶100 mM NH_4_COOH(aqueous) 58∶40∶2 (mobile phase A). Quantification of glycerophospholipids was achieved by the use of an LC-MS technique employing synthetic odd-carbon diacyl and lysophospholipid standards. Typically, 200 ng of each odd-carbon standard was added per sample. Glycerophospholipids were analyzed on an Applied Biosystems/MDS SCIEX 4000 Q TRAP hybrid triple quadrupole/linear ion trap mass spectrometer (Applied Biosystems) and a Shimadzu high pressure liquid chromatography system with a Phenomenex Luna Silica column (5-µm particle size) using a gradient elution as previously described [35], [36]. Individual species were identified based on their chromatographic and mass spectral characteristics. This analysis allows identification of the two fatty acid moieties but does not determine their position on the glycerol backbone (sn-1 versus sn-2). Neutral lipids from zebrafish (forty of 8 dpf larvae/sample) were extracted by homogenization in the presence of internal standards (500 ng 14∶0 monoacylglycerol and 24∶0 diacylglycerol and 1 µg 42∶0 triacylglycerol) in 2 ml 1× PBS and extracting with 2 mL ethyl acetate∶trimethylpentane (25∶75). A dried lipid film was dissolved in 1 mL hexan∶sopropanol (4∶1) and passed through a bed of Silica gel 60 Å to remove remaining polar phospholipids. Solvent from the collected fractions was evaporated and lipid film was redissolved in 90 µl 9∶1 CH_3_OH∶CHCl_3_, containing 10 µl of 100 mM CH_3_COONa for MS analysis essentially as previously described [36], [37]. Samples were analyzed in triplicates and p-values determined using Student's *t*-test.

### Acylcarnitine and Organic Acid Profiling

Forty 9 dpf control and *dxa* mutant larvae were lysed using pellet pestles (Sigma, #Z359947) and passed through a 25 gauge syringe in 150 µL of PBS. For acylcarnitine analysis, the total lysate was placed into a 96 well plate containing stable isotope labeled internal standards (Cambridge Isotope Laboratories, Andover, MA) and acylcarnitine analysis performed according to the published methods [38]. Briefly, the lysate was dried under nitrogen, reconstituted with fifty µL of acetonitrile and one µL was injected into a Xevo-TQS tandem mass spectrometer (Waters Corp. Waltham, MA). Acylcarnitines were quantified against an isotope–labeled internal standard of the nearest chain-length using the parent ions of the carnitine-specific fragment of m/z 85. For organic acid analysis, the total lysate was made up to a final volume of 2.5 mL using deionized water, acidified to pH 2.0 and the acid fraction extracted three times into equal volumes of ethyl acetate. The pooled organic phases were dried down under a stream of nitrogen at room temperature and trimethylsilyl derivatives analyzed by gas chromatography-mass spectrometry using an Agilent 7890A gas chromatograph fitted with a 5975C Mass Selective Detector (Agilent Technologies, Santa Clara, CA) using a method initially developed for urine and vitreous humor analysis [39]. The acylcarnitine and organic acid assays are validated and in routine clinical use and were also previously used for analyses of *etfdh* mutant zebrafish [12].

### Transmission Electron Microscopy

In brief, samples were fixed in 2.5% gluteraldehyde for 1 hour then transferred to 4°C overnight. Samples were washed 3 times in 0.1 M cacodylate buffer then incubated for 1 hour in 1% osmium tetraoxide and washed with cacodylate buffer. Samples were dehydrated through a graded series of ethanol, then incubated in ethanol and propylene oxide (PO). Samples were infiltrated with 25% Epon 812 resin and 75% PO for 35 minutes, then 50% Epon 812 resin and 50% PO for 1 hour then exchanged with new 50% Epon 812 resin and 50% PO and incubated overnight. Samples were exchanged with 75%: 25% (resin: PO), then pure epoxy resin for 3–4 hours, then overnight. Finally, the resin was exchanged with epoxy resin for 3 hours, embedded in epoxy resin and polymerized at 60°C for 48 hours.

*Sectioning and Imaging*: 500 nm to 1 µm thick sections were collected using a Leica Ultracut microtome. Thick sections were stained with 1% toluidine blue and. 70–80 nm ultra-thin sections were cut from this block and collected on 300-mesh copper grids and stained with 2% uranyl acetate (aqueous) for 16 minutes and then with lead citrate for 12 minutes. Samples were imaged on the Philips/FEI Tecnai T12 electron microscope at various magnifications.

### High Resolution Respirometry

Average basal oxygen flux was quantified by high resolution respirometry using the Oroboros O2k Oxygraph (Oroboros Instruments, Innsbruck, Austria). Ten larvae (8 dpf) per chamber were maintained in Instant Ocean at 28°C and initially equilibrated to room air. Measurements of oxygen concentration were recorded every 2 seconds with no stirring. When the measured O_2_ concentration stabilized, chambers were stirred at 100 rpm for as short a time as possible to permit recording of a new stable O_2_ concentration, which was reflective of the true O_2_ concentration in solution. Stirrers were then turned off. This process was repeated until no further decrement in O_2_ concentration was measured and the fish were no longer motile. Average oxygen flux was calculated from the change in O_2_ concentration over time from the beginning of the experiment to the end. Data are from 4 measurements made with 10 larvae of each genotype.

### Leucine Analysis

Forty control siblings and homozygote *dxa* mutant larvae from 3 to 5 clutches were homogenized in 100–750 µL of 0.1 M TCA, containing 10 mM sodium acetate, 100 µM EDTA, 5 ng/ml isoproterenol as an internal standard and 10.5% methanol at pH 3.8. Samples were centrifuged at 10,000× g for 20 minutes. Supernatant was removed and stored at −80 degrees. Samples of the supernatant were then analyzed for biogenic monoamines and/or amino acids. Leucine was quantified with a Waters AccQ-Tag system with a Waters 474 Scanning Fluorescence Detector. Ten µL samples of the supernatant are diluted with 70 µL of borate buffer to which 20 µL aliquots of 6-Aminoquinol-N-Hydroxysuccinimidyl Carbamate and 10 µL 250 pmol/µL α-aminobutyric acid (as internal standard) are added to form fluorescent derivatives. 10 µL of sample was then injected into the HPLC system, and separation of the amino acids accomplished by means of a Waters amino acid column and supplied buffers using a specific gradient profile.

### Statistical Analysis

Error bars in Figure S3 and S6 represent standard error of the mean (SEM), error bars in Figure 7 and S4 represent standard deviations (SD). Student's t-test was used to determine statistical significance.

### Ethics Statement

All animal experiments were done with the approval of the Vanderbilt University IACUC.

## Supporting Information

Figure S1 Whole-mount in situ hybridization of *etfa* during development. (A) Expression of *etfa* during early development. (B) Comparison of *etfa* expression in wild type and homozygous *dxa* mutant at 30 hours post fertilization. Mib, midbrain; bv, blood vessel; PF, pectoral fin; L, liver. Scale bar = 100 µm. (C) No expression of Etfa protein in WT and *dxa* mutant zebrafish at 6 dpf.
(TIF)

Figure S2 Abnormal acylcarnitine and organic acids in *dxa^vu463^* mutant zebrafish. (A) Representative profile from homogenized control siblings and homozygous *dxa* mutant larvae (9 dpf) using tandem mass spectrometry. The amount of species (µmol) is shown on the top of bars. (B) Organic acid profile from control siblings and (C) *dxa* mutant (9 dpf) using gas chromatography electron impact mass spectrometric analysis. Arrows indicate glutaric acid. N = 40 for both control siblings and homozygous mutant *dxa* zebrafish.
(TIF)

Figure S3 Liver defects and increased size of hepatocytes in *dxa^vu463^* mutant zebrafish. (A,B) ORO and DAPI staining of wild type (A) and *dxa* mutant liver at 6 dpf (B). (C,D) Filipin staining of wild type (C) and *dxa* liver at 6 dpf (D). Each of single cells are outlined with yellow in A–D, magnified views in lower left corner (C–D) shows absence of cholesterol accumulation in the cytosol of *dxa*. (E,F) Filipin staining of sibling control (E) and type III *dxa* (F) for cell size measurement at 8 dpf. Representative cells used for analysis are outlined in blue. (G) Total pixel numbers were measured to compare relative cell size in selected areas in E and F. Thirty cells (10 cells/larvae) were measured and control and mutant size were compared, p<0.001. Scale bar = 50 µm (A,B) and 100 µm (C–F).
(TIF)

Figure S4 Decreased oxygen flux in type III *dxa^vu463^* at 8 dpf. Oxygen flux was measured by comparing oxygen consumption over time in control siblings and homozygous *dxa^vu463^* mutants. After the measured O_2_ concentration in the Oroboros O2k Oxygraph was stabilized, chambers were stirred at 100 rpm to allow recording of a new stable O_2_ concentration. Stirrers were then turned off as constant stirring was deleterious to the zebrafish larvae. This process was repeated until no further decrement in O_2_ concentration was measured and the fish were no longer motile. Average oxygen flux was then calculated from the change in O_2_ concentration over time from the beginning of the experiment to the end. p<0.007, Each experiment was repeated 4 times using 10 larvae of each genotype for each measurement.
(TIF)

Figure S5 Increased numbers of neural progenitor cells in *dxa^vu463^* mutant brain. (A) Sox2 positive cells in the VZ were counted in sibling control (WT) and type II *dxa* mutants at 8 dpf. (B) Quantitative comparison of Sox2 positive cell numbers between WT siblings and type II *dxa* mutant. Scale bar = 100 µm, n = 3 larvae, p  = 0.01.
(TIF)

Figure S6 Lipid analysis in *dxa^vu463^* at 8 dpf. (A) Total monoacylglycerol (MAG), diacylglycerol (DAG) and triacylglycerol (TAG) level were compared with MAG decreases and TAG increases being statistically significant, *p<0.01, **p<0.001, n = 3. (B) Total phosphatidylcholine (PC) levels were unchanged with a slight statistically significant increase in phosphatidylethanolamine (PE), p = 0.043 and a marked statistically significant decrease in phosphatidylserine (PS) p<0.001, n = 3.
(TIF)

Figure S7 mTORC1 activation in *dxa^vu463^* at 6 dpf. (A) Immunoblot blots for phospho-S6Ser235/236 and phosphor-4E-BP1Thr37/46 in control sibling and *dxa* mutant zebrafish at 6 dpf. (B) Relative ratio of phospho-S6 amount in control and *dxa* mutants, p = 0.0003. (C) Relative ratio of phosphor-4E-BP1 amount in control and *dxa* mutants. P = 0.035, n = 5 samples per genotype, each samples contained 2 larvae. p = 0.0246. (D) Phospho-S6 (left panel) and phospho-4E-BP1 staining (right panel) in the liver of WT (top) and type II *dxa* (bottom) zebrafish at 6 dpf. Scale bar = 100 µm.
(TIF)

Figure S8 Increased leucine levels in *dxa^vu463^* at 8 dpf. Leucine was quantified using HPLC. Extracts of 40 siblings and 40 grouped type II and III homozygous larvae were measured and levels compared. Average amount of leucine is indicated on the top of each bar. Larvae were collected from 3 to 5 clutches for each experiment. p = 0.05, n = 3.
(TIF)
