# Supplementary figures and images for: Multi-organ Abnormalities and mTORC1 Activation in Zebrafish Model of Multiple Acyl-CoA Dehydrogenase Deficiency
Source: PLoS Genet. 2013 Jun 13;9(6):e1003563. doi: 10.1371/journal.pgen.1003563 (PMC3681725; doi:10.1371/journal.pgen.1003563)

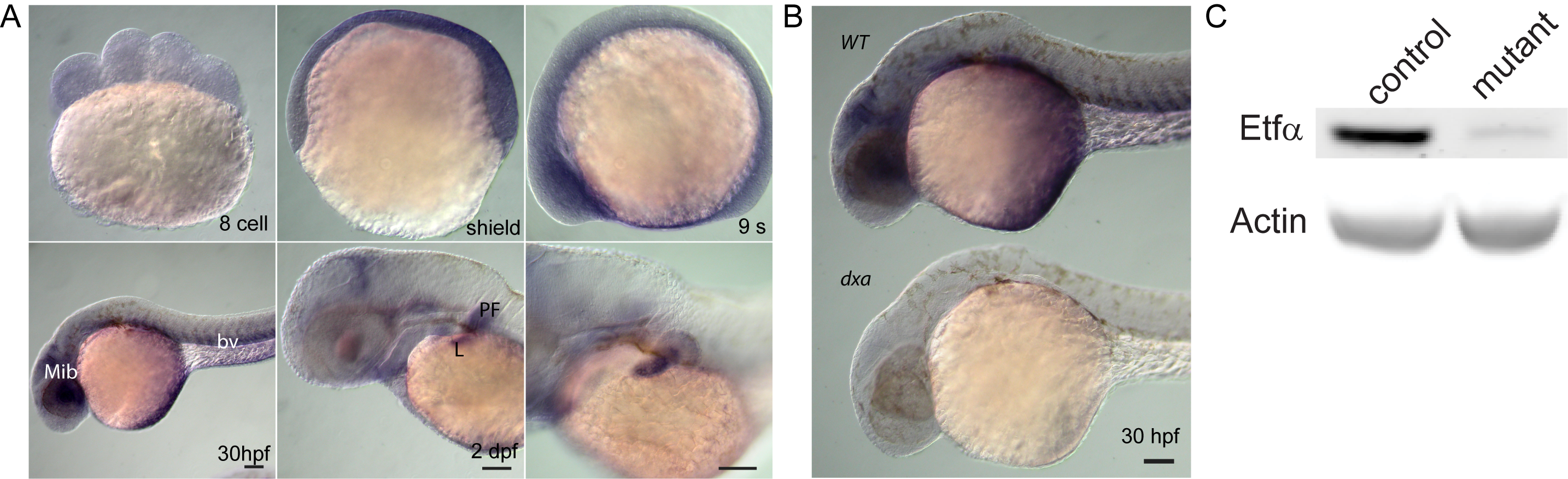

Supplement: Figure S1 — Whole-mount in situ hybridization of etfa during development. (A) Expression of etfa during early development. (B) Comparison of etfa expression in wild type and homozygous dxa mutant at 30 hours post fertilization. Mib, midbrain; bv, blood vessel; PF, pectoral fin; L, liver. Scale bar = 100 µm. (C) No expression of Etfa protein in WT and dxa mutant zebrafish at 6 dpf. (TIF) [file pgen.1003563.s001.tif]

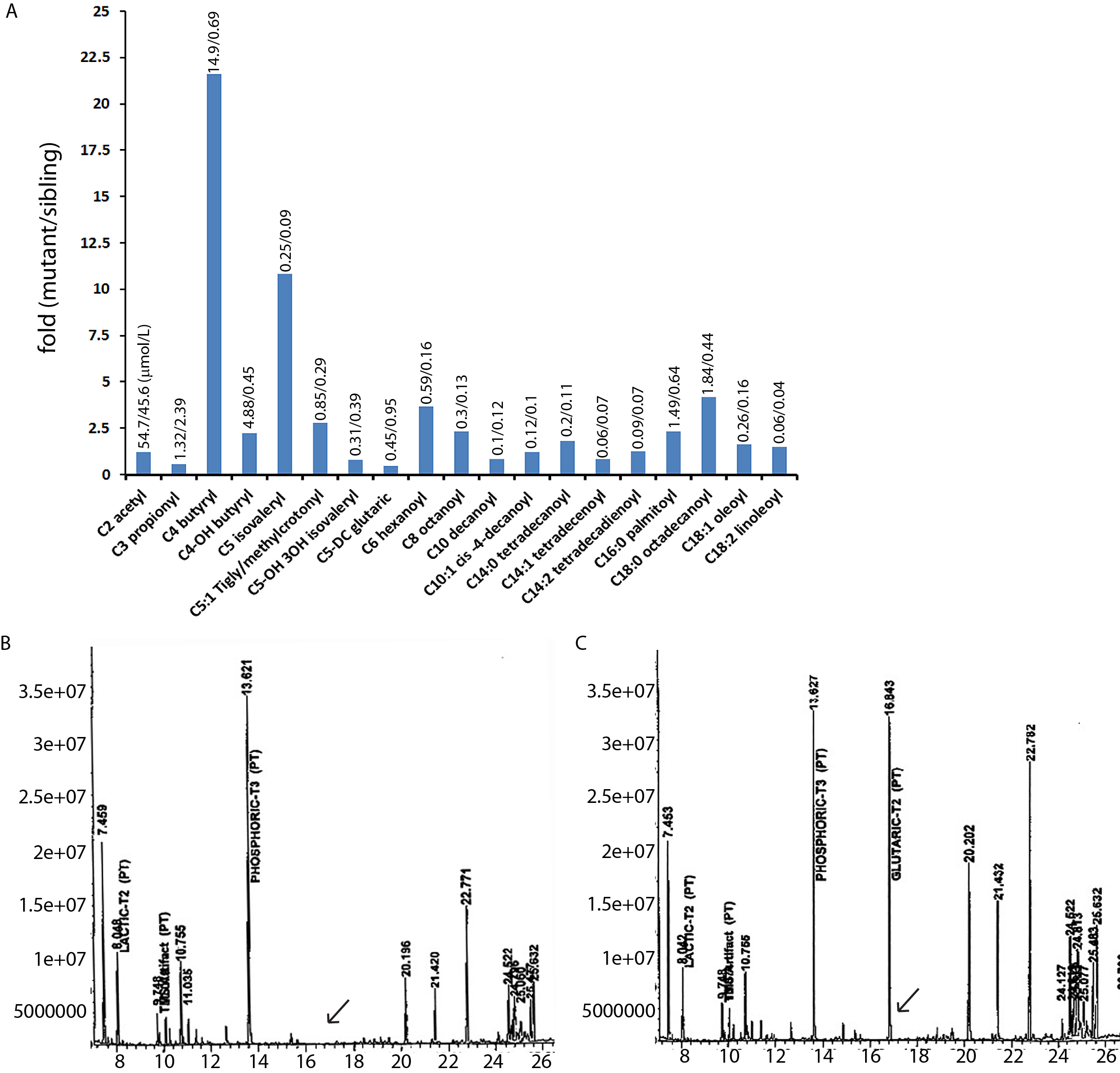

Supplement: Figure S2 — Abnormal acylcarnitine and organic acids in dxavu463 mutant zebrafish. (A) Representative profile from homogenized control siblings and homozygous dxa mutant larvae (9 dpf) using tandem mass spectrometry. The amount of species (µmol) is shown on the top of bars. (B) Organic acid profile from control siblings and (C) dxa mutant (9 dpf) using gas chromatography electron impact mass spectrometric analysis. Arrows indicate glutaric acid. N = 40 for both control siblings and homozygous mutant dxa zebrafish. (TIF) [file pgen.1003563.s002.tif]

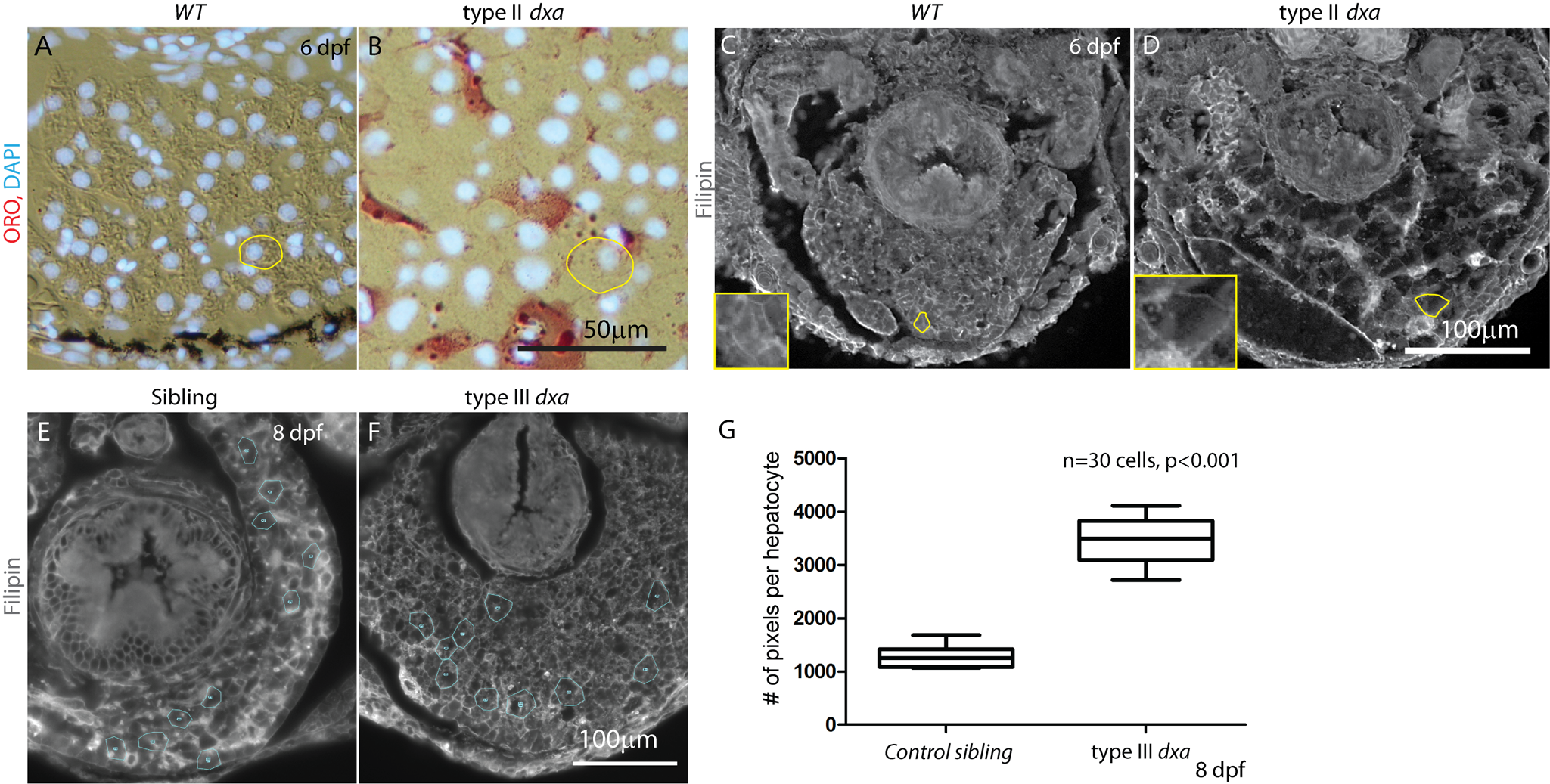

Supplement: Figure S3 — Liver defects and increased size of hepatocytes in dxavu463 mutant zebrafish. (A,B) ORO and DAPI staining of wild type (A) and dxa mutant liver at 6 dpf (B). (C,D) Filipin staining of wild type (C) and dxa liver at 6 dpf (D). Each of single cells are outlined with yellow in A–D, magnified views in lower left corner (C–D) shows absence of cholesterol accumulation in the cytosol of dxa. (E,F) Filipin staining of sibling control (E) and type III dxa (F) for cell size measurement at 8 dpf. Representative cells used for analysis are outlined in blue. (G) Total pixel numbers were measured to compare relative cell size in selected areas in E and F. Thirty cells (10 cells/larvae) were measured and control and mutant size were compared, p<0.001. Scale bar = 50 µm (A,B) and 100 µm (C–F). (TIF) [file pgen.1003563.s003.tif]

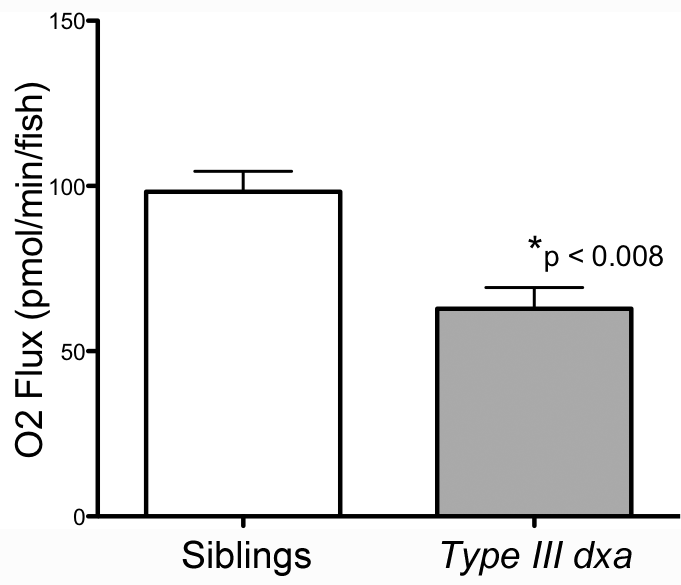

Supplement: Figure S4 — Decreased oxygen flux in type III dxavu463 at 8 dpf. Oxygen flux was measured by comparing oxygen consumption over time in control siblings and homozygous dxavu463 mutants. After the measured O2 concentration in the Oroboros O2k Oxygraph was stabilized, chambers were stirred at 100 rpm to allow recording of a new stable O2 concentration. Stirrers were then turned off as constant stirring was deleterious to the zebrafish larvae. This process was repeated until no further decrement in O2 concentration was measured and the fish were no longer motile. Average oxygen flux was then calculated from the change in O2 concentration over time from the beginning of the experiment to the end. p<0.007, Each experiment was repeated 4 times using 10 larvae of each genotype for each measurement. (TIF) [file pgen.1003563.s004.tif]

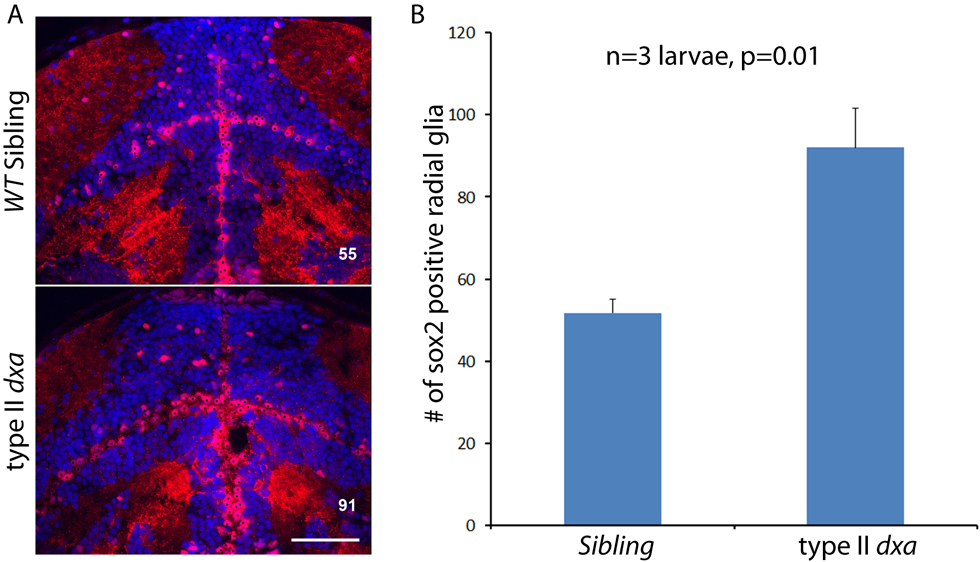

Supplement: Figure S5 — Increased numbers of neural progenitor cells in dxavu463 mutant brain. (A) Sox2 positive cells in the VZ were counted in sibling control (WT) and type II dxa mutants at 8 dpf. (B) Quantitative comparison of Sox2 positive cell numbers between WT siblings and type II dxa mutant. Scale bar = 100 µm, n = 3 larvae, p = 0.01. (TIF) [file pgen.1003563.s005.tif]

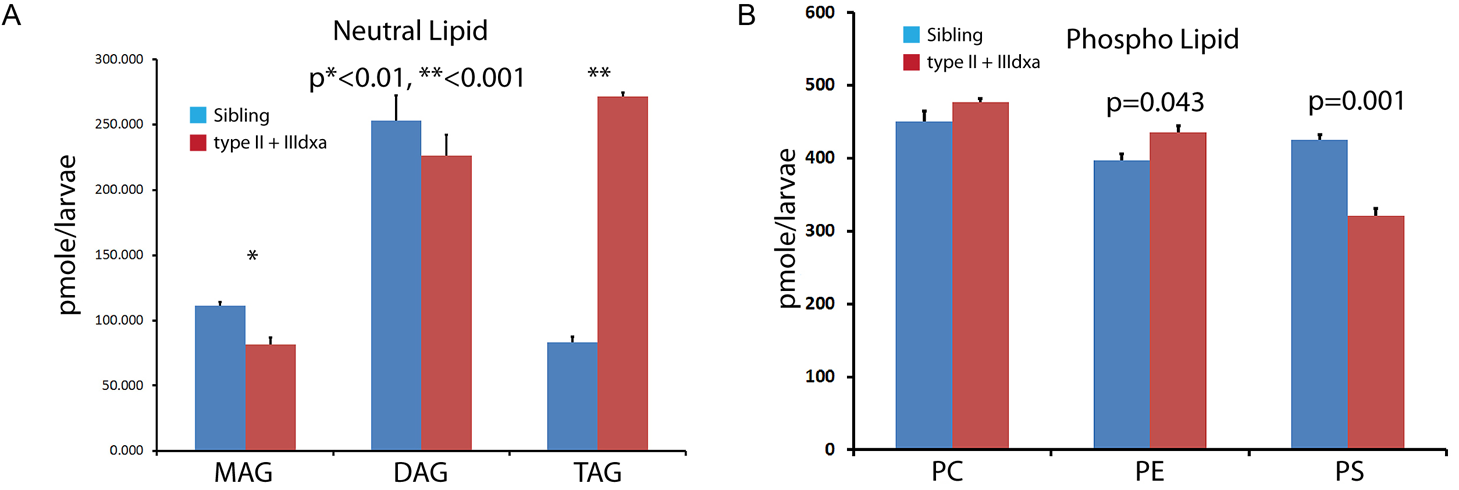

Supplement: Figure S6 — Lipid analysis in dxavu463 at 8 dpf. (A) Total monoacylglycerol (MAG), diacylglycerol (DAG) and triacylglycerol (TAG) level were compared with MAG decreases and TAG increases being statistically significant, *p<0.01, **p<0.001, n = 3. (B) Total phosphatidylcholine (PC) levels were unchanged with a slight statistically significant increase in phosphatidylethanolamine (PE), p = 0.043 and a marked statistically significant decrease in phosphatidylserine (PS) p<0.001, n = 3. (TIF) [file pgen.1003563.s006.tif]

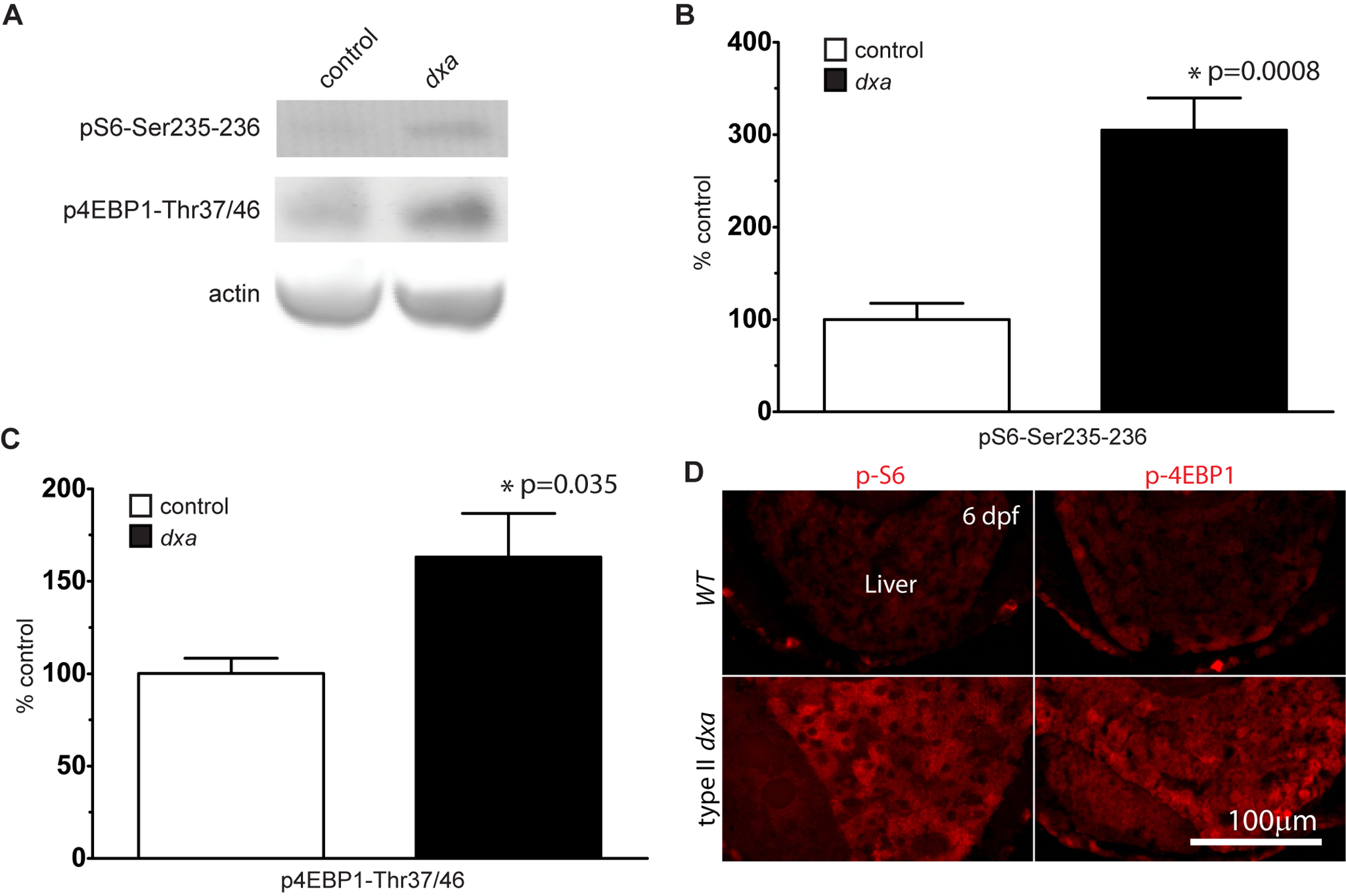

Supplement: Figure S7 — mTORC1 activation in dxavu463 at 6 dpf. (A) Immunoblot blots for phospho-S6Ser235/236 and phosphor-4E-BP1Thr37/46 in control sibling and dxa mutant zebrafish at 6 dpf. (B) Relative ratio of phospho-S6 amount in control and dxa mutants, p = 0.0003. (C) Relative ratio of phosphor-4E-BP1 amount in control and dxa mutants. P = 0.035, n = 5 samples per genotype, each samples contained 2 larvae. p = 0.0246. (D) Phospho-S6 (left panel) and phospho-4E-BP1 staining (right panel) in the liver of WT (top) and type II dxa (bottom) zebrafish at 6 dpf. Scale bar = 100 µm. (TIF) [file pgen.1003563.s007.tif]

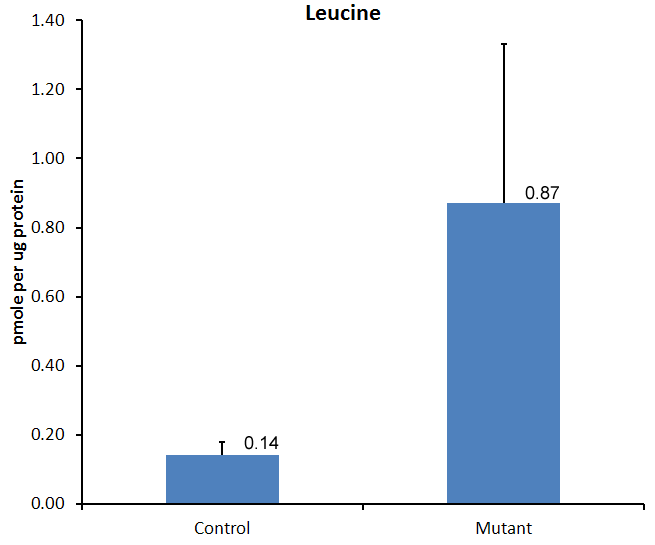

Supplement: Figure S8 — Increased leucine levels in dxavu463 at 8 dpf. Leucine was quantified using HPLC. Extracts of 40 siblings and 40 grouped type II and III homozygous larvae were measured and levels compared. Average amount of leucine is indicated on the top of each bar. Larvae were collected from 3 to 5 clutches for each experiment. p = 0.05, n = 3. (TIF) [file pgen.1003563.s008.tif]
